# Solid State Joining of a Cold Rolled Zr-Based Bulk Metallic Glass to a Wrought Aluminum Alloy by Power Ultrasonics

**DOI:** 10.3390/ma15217673

**Published:** 2022-11-01

**Authors:** Michael Becker, Alexander Kuball, Amirhossein Ghavimi, Bastian Adam, Ralf Busch, Isabella Gallino, Frank Balle

**Affiliations:** 1 Walter-and-Ingeborg-Herrmann-Chair for Power Ultrasonics and Engineering of Functional Materials (EFM), Department of Sustainable Systems Engineering (INATECH), Faculty of Engineering, University of Freiburg, Emmy-Noether-Str. 2, 79110 Freiburg i.Br., Germany; 2 Amorphous Metal Solutions GmbH, Michelinstraße 9, 66424 Homburg, Germany; 3 Chair of Metallic Materials, Department of Materials Science and Engineering, Faculty of Natural Sciences and Technology, Saarland University, Campus C6.3, 66123 Saarbrücken, Germany; 4 Freiburg Materials Research Center (FMF), Stefan-Meier-Str. 21, 79104 Freiburg i.Br., Germany; 5 Fraunhofer Institute for High-Speed Dynamics, Ernst-Mach-Institut, EMI, Ernst-Zermelo-Str. 4, 79104 Freiburg i.Br., Germany

**Keywords:** ultrasonic metal welding, aluminum alloy sheet, zirconium-based bulk metallic glass, amorphous/crystalline joint

## Abstract

Ultrasonic metal welding (UMW) enables joining in the solid state at relative low temperatures with short cycle times. This technique is of particular interest for joining metallic glasses to each other or to other materials, because crystallization of the amorphous structure can be prevented due to the low thermal loading and the rapidity of the process. In this work, UMW is applied to join one 1 mm thick sheet of a commercial wrought aluminum alloy (AA5754) and one 0.4 mm thick strip of a commercial Zr-based bulk metallic glass (AMZ4). The introduced heat of the welding process is detected with thermocouples and thermal imaging. To investigate the strength of the joint and the influence on the microstructure, mechanical tensile tests are carried out in combination with scanning electron microscopy and differential scanning calorimetry. The results show that ultrasonic metal welding is a suitable technique to join amorphous bulk metallic glasses to crystalline aluminum alloys. The metallic glass component retains its amorphous structure in the joint, and the joint strength is higher than the strength of the Al sheet. These findings will help to develop future applications of BMG-based multi-material components, including medical tools.

## 1. Introduction

Bulk metallic glasses (BMGs) differ significantly from crystalline metals in their mechanical, physical and chemical properties due to their amorphous structure [1,2,3,4]. For example, BMGs have extraordinarily high hardness values (in the range of 350 up to 800 HV1), as well as high strength (2 to 4 GPa of yield strength) and excellent wear and corrosion resistance [2,5,6,7]. The exceptional combination of these properties makes BMGs attractive for use as an engineering material for a variety of applications. The development of expanded areas of application requires the possibility of joining with materials of the same type and, in particular, with crystalline materials, including light alloys such as aluminum [3,4]. In addition to the direct manufacture of a product, the production of semi-finished products is also possible, for example composite materials in sandwich construction with a crystalline core and metallic glasses as cover layers [8]. A suitable joining technique must ensure that, in addition to high joint strength, the amorphous structure of the metallic glasses and thus their special properties are retained [7]. It is therefore particularly important that the heat input during the joining process is as low and short as possible to avoid crystallization. Therefore, the maximum process temperature has to be below the crystallization temperature, which is a function of time. Ultrasonic (US) welding meets this demand due to short cycle times as well as low and local heat exposure of the materials [9,10]. Further advantages of ultrasonic metal welding include energy efficiency and no need for welding consumables [11]. Another essential feature is the ability to join materials with different melting temperatures, since the process takes place in the solid state [12].

Some studies have already been reported on the use of ultrasonic welding technology in the processing of bulk metallic glasses. For example, the ultrasonic plastic welding technique has been successfully used for thermoplastic forming [1] as well as rejuvenation of BMGs to reduce their brittleness and improve their plasticity [13,14]. Ultrasonic plastic welding joined metallic glasses with thicknesses from 0.04 mm up to 2 mm to each other [15,16,17,18]. Suitable process parameters resulted in entirely welded interfaces [16], and further layers could be added separately on top [15]. The base materials as well as the interfaces remained in the amorphous state. In addition, ultrasonic metal welding was applied to produce bulk metallic glasses by joining metallic glass foils with thicknesses from 0.02 mm to 0.05 mm [19,20,21,22]. The thermal properties of the foils were preserved [20], and bulk metallic glass was formed without crystallization. Maeda et al. [23] reported investigations of Zr_55_Cu_30_Ni_5_Al_10_ at.% metallic glass joints and found that even a small temperature overshooting above the glass transition temperature during ultrasonic welding did not crystallize the amorphous structure. There are also reports of ultrasonic welding of crystalline metals to amorphous metallic glasses [24,25]. Kreye et al. [26] successfully welded 0.8 mm thick copper strips to 0.05 mm thick ribbons of Ni_40_Fe_40_P_14_B_6_ at.% metallic glass. There was no crystallization of the metallic glass due to the very short heating time. Nishikawa et al. [4] bonded a pure Al (99.99%) wire with 0.2 mm diameter on Cu-, Ni- and Zr-based BMGs and achieved straight as well as sharp interfaces without visible intermetallic phases. Li et al. [27] used the ultrasonic plastic welding technique to join 0.1 mm thick Al and Cu ribbons to 0.04 mm thick Ni_82_._2_Cr_7_B_3_Si_4_._8_Fe_3_ at.% metallic glass ribbons. They found no intermetallic phases at the interfaces and no crystallization of the metallic glass.

Based on the promising results of the applications of ultrasonic welding of amorphous metallic glasses, especially in combination with crystalline metals, at relatively small material thicknesses, the transition to larger material thicknesses is a possible option. This is relevant with regard to structural applications that, for example, require larger cross-sections to meet strength requirements due to mechanical loads. Therefore, the crystalline joining partner in this investigation is a commercial aluminum alloy used as structural material. Since the properties of ultrasonic metal welding are strongly influenced by the geometry and, in particular, the thickness of the joining partners, the results of the investigations with relatively thin joining partners cannot be simply scaled. Thus, new studies need to be conducted using the materials with corresponding geometries in order to gain knowledge about the feasibility and process properties. To the authors’ knowledge, the first ultrasonically welded joint of a commercial BMG with an aluminum alloy on a macroscopic scale, suitable for determining the mechanical properties of the joint in a tensile shear test, is presented. Measurements by a thermocouple at the interface and an infrared camera on top of the aluminum sheet reveal the temperature development during the welding process. The study is intended to support the transition from the laboratory to the component level and to expand the world of weldable BMGs to conventional engineering alloys. Possible areas of application are, in particular, special tools, for example, in medical technology.

## 2. Materials and Methods

**Figure 1:**
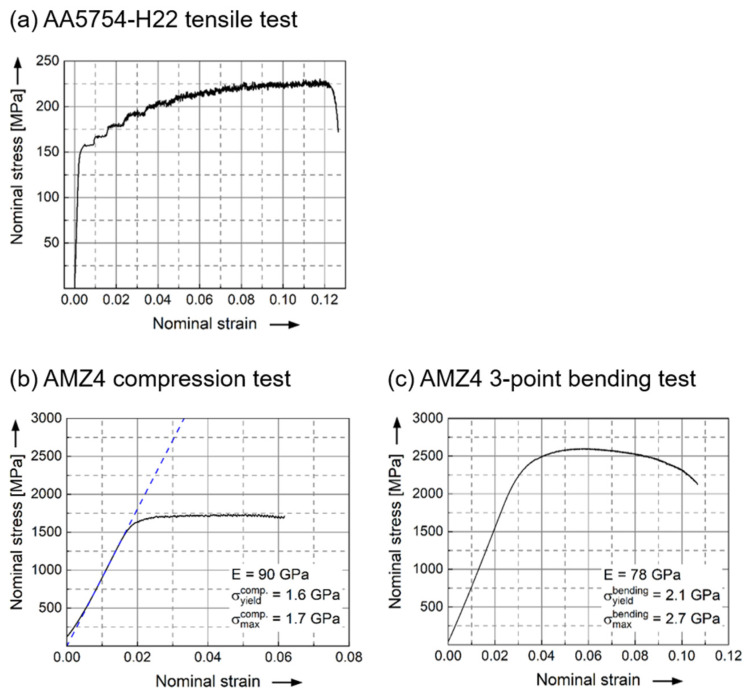
Representative curves showcasing the data from Table 1. (**a**) Tensile tests of AA5754-H22 according to the DIN EN ISO 6892-1 process A, (**b**) compression and (**c**) 3-point bending test of AMZ4.

The commercial aluminum alloy AA5754 (with atomic composition Al_96.447_Si_0.179_Mg_3.096_Fe_0.135_Cu_0.008_Mn_0.112_Cr_0.01_Zn_0.008_Ti_0.005_ at.%) and the commercial amorphous metal with atomic composition Zr_59.3_Cu_28.8_Al_10.4_Nb_1.5_ at.% (named AMZ4 [28] and trademarked by Heraeus AMLOY, Karlstein, Germany, as ZR01) are used in this investigation. The aluminum wrought alloy was supplied by Hydro Aluminium (Bonn, Germany) in the form of sheets with dimensions 70 mm × 20 mm × 1 mm in condition H22 (work-hardened by rolling, final annealing to quarter hard condition), with Vickers hardness of 72 ± 1 HV1. This alloy is typically used for rivets, welded chemical and nuclear structures, ship building and vehicle bodies. The metallic glass strips of AMZ4 were produced at the chair of metallic materials at Saarland University in a three-step process. To produce the master alloy, high purity raw elements are melted and mixed in an electric arc furnace under a high purity argon atmosphere. Subsequently, the master alloy is remelted in a custom-built suction casting device and is cast into a plate shaped water-cooled copper mold. The dimensions of the as-cast plates are 10 mm × 40 mm × 2 mm. Finally, the as-cast plates are cold rolled down to a thickness of 0.4 mm, resulting in strips of the length of about 200 mm. The strips achieve a Vickers hardness of 450 ± 20 HV5 and are cut to approximately 55 mm long sections for the welding trials. Table 1 shows mechanical properties parameters for each of the two alloys, and the underlying representative curves are provided in Figure 1.

**Figure 2:**
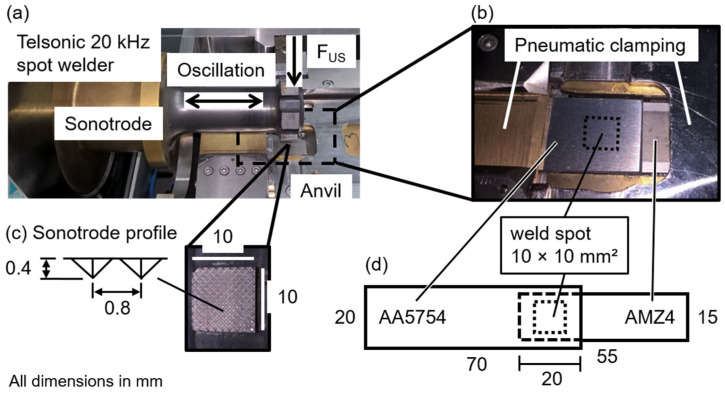
(**a**) The Telsonic spot welder, oscillating at 20 kHz, with custom-made anvil for positioning and clamping of AA5754 and AMZ4. (**b**) AA5754 and AMZ4 clamped separately before welding; the dotted square indicates the position of the weld spot. (**c**) Details of the square 10 × 10 mm^2^ sonotrode profile with pyramids of 0.4 mm height and 0.8 mm distance from each other. (**d**) Geometry of the overlapping specimens with AA5754 on the tool side.

The spot welding system consists of a commercially available Telsonic (Bronschhofen, Switzerland) longitudinal welding system (type M4000) with a maximum power output of 6000 W, equipped with a custom-made anvil, as shown in Figure 2a. The welding tool, the so-called sonotrode, oscillates at 20 kHz in the direction of the symmetry axis. The squared coupling surface of the tip, measuring 10 × 10 mm^2^, is shaped with a pyramidal profile, as shown in Figure 2c. The pyramidal profile is aligned perpendicular to the oscillation direction. The profile ensures a better transmission of the ultrasonic oscillation and reduces relative movement between the sonotrode and the sheet on the tool side. The welding force F_US_ is generated pneumatically up to 4000 N and presses the sonotrode onto the sheets placed on the anvil during welding. The anvil offers the possibility to clamp both joining partners separately (Figure 2b). All contact surfaces are flat, and the clamping effectively prevents unwanted movements of the sheets, especially of the joining partner on the anvil side. An integrated force measurement system monitors the welding force F_US_.

AA5754 is used as the upper sheet for all experiments since this leads to less wear at the coupling surface compared to when the harder metal is used on the tool side. The surface roughness of the joining partners is determined by holography, using a Fraunhofer IPM (Institute for Physical Measurement Techniques, Freiburg i.Br., Germany) HoloTop 65M18 [29,30]. The average roughness value Ra is 0.45 ± 0.03 µm for AA5754 and 0.59 ± 0.04 µm for AMZ4. Parallel long edges align the aluminum sheet and AMZ4 strip in rolling direction, and the overlap length is 20 mm (marked by a dotted line in Figure 2d). The weld spot is created in the middle of the overlap area. Complex pre-treatments of the materials are avoided to emulate the testing conditions as close as possible to a potential application. The sheets and strips are cleaned using ethanol prior to welding to ensure the removal of dirt or grease and thus the consistency of the initial conditions [31].

During certain welding tests, temperature profiles are recorded using a Micro-Epsilon (Ortenburg, Germany) TIM infrared camera with a microscope lens at 32 frames per second. The working distance is approx. 10 mm, and the optics are focused on the gap between the tool and the aluminum sheet. Since it is a very narrow gap, an emissivity value of 1 is used. The measurement settings are checked in preliminary tests with a thermocouple at the appropriate location. Tensile shear tests are performed at a constant speed of 0.016 mm/s at room temperature (standard atmosphere) using a ZwickRoell (Ulm, Germany) Z020 universal testing machine. Cross-sections of welded samples are prepared and microscopically examined using the Zeiss (Oberkochen, Germany) Smartzoom 5 digital optical microscope and characterized in the Zeiss EVO 15 scanning electron microscope (SEM) equipped with energy-dispersive X-ray spectroscopy (EDX). X-ray diffraction analysis (XRD) of material surfaces as well as fractured surfaces are performed using monochromatic CuKα radiation at λ = 1.54 Å with a Malvern Panalytical (Malvern, UK) X’Pert^3^ MRD system at INATECH.

Calorimetric measurements are performed using a PerkinElmer (Beaconsfield, UK) DSC8000 with samples of approximately 20 mg of mass, which are enclosed in Al-pans and scanned with a heating rate of 0.33 K/s from 323 to 853 K under a constant high purity argon flux. The calorimeter is calibrated for the applied measuring conditions and for the applied pans (alternatively Al and Cu) for temperature and enthalpy of reaction using standards, i.e., In, Zn. In order to obtain a baseline for each specimen the measurement is repeated in a second up-scan under identical conditions with the crystallized alloy, without removing the sample. The second heat flow scan is subtracted from the first scan to obtain a flat baseline.

## 3. Results and Discussion

In the energy-controlled approach shown in this investigation, the main welding parameters are the welding energy (W_US_), the displacement amplitude (u) and the welding force (F_US_). The tensile shear force (F_TS_) is obtained in a tensile test of the joint and is considered to be the main quality criterion for the single overlapped Al/BMG joints, with the aim of creating quantitatively reliable and reproducible weld quality and strength. The values of the process parameters are systematically increased between the welding tests to find suitable values. A total of 44 welds covers the parameter ranges of the welding energy from 1000 to 2200 Ws, the displacement amplitude from 26 to 44.2 µm and the welding force from 130 to 790 N. Since AA5754 is the joining partner on the tool side, the sonotrode has to transmit both the oscillations and the welding force via the aluminum to the interface. The oscillations at the interface must be large enough to generate a relative movement between the two joining partners [32,33]. In combination with a sufficient welding force that presses the joining partners together, oxide layers that can hinder a joint can be broken up and displaced from the weld zone [34]. Furthermore, ultrasonic metal welding requires sufficient ductility of at least one joining partner at the interface to produce a joint in the solid state. Microscopic roughness peaks are sheared off and flattened in order to provide a large surface area of contact that leads to the formation of covalent bonds [35,36].

**Figure 3:**
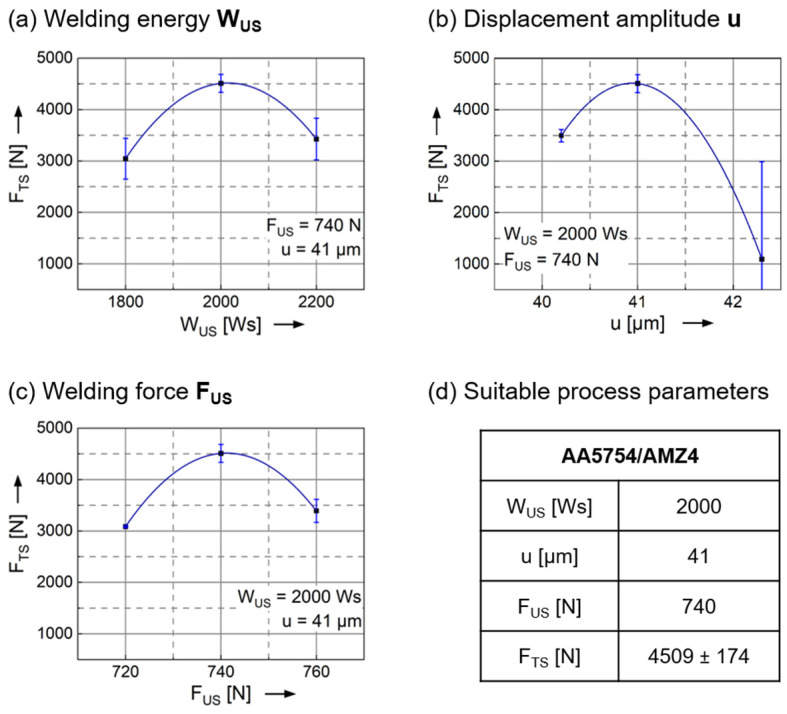
(**a**) Influence of the welding energy W_US_ on the tensile shear force F_TS_, welding force and displacement amplitude are kept constant. (**b**) Influence of the displacement amplitude u on the tensile shear force F_TS_, welding energy and welding force are kept constant. (**c**) Influence of the welding force F_US_ on the tensile shear force F_TS_, welding energy and displacement amplitude are kept constant. Each diagram is based on nine trials. (**d**) Suitable process parameters for AA5754/AMZ4 joints with the highest tensile shear forces and the best reproducibility.

**Figure 4:**
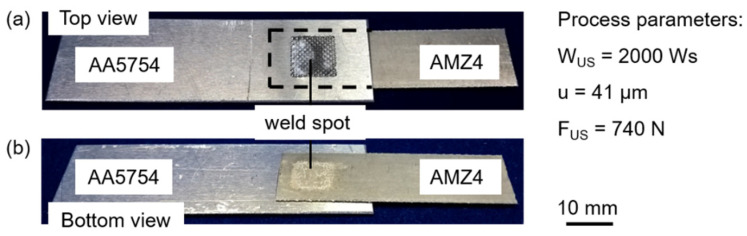
(**a**) Top view of a welded specimen showing a typical imprint of the sonotrode surface with the pyramidal profile. (**b**) Bottom view of a welded specimen showing a slight discoloration in the area of the weld spot. Process parameters: welding energy W_US_ = 2000 Ws, displacement amplitude u = 41 µm and welding force F_US_ = 740 N.

The combination of the relatively soft AA5754 and the around 10 times stronger AMZ4 requires a high amount of energy to enable plastic deformation at the interface [37,38]. In addition, process parameters with large values of the displacement amplitude and small ones of the welding force result in large plastic strains in the welding zone and enhance joint formation [37]. A displacement amplitude lower than 38 µm and a welding force lower than 700 N does not result in bonded materials. Higher displacement amplitudes than 41 µm usually cause deep imprints of the sonotrode surface pattern on the low-hardness aluminum sheets. These deep indentations often lead to cracks in the Al sheet at the edges and are sometimes seen to grow into the adjacent material as well. These cracks are observed to fail prematurely during tensile testing, resulting in a significant increase in the scatter of the tensile shear strength. Thus, as a result of our study, the applicable ranges for each parameter are found to be significantly narrowed. Starting from the parameter combination with the highest tensile forces, each of the three main parameters is varied individually. Figure 3 shows the results of this approach. Each diagram contains the influence on the tensile shear force F_TS_ of the variation of one parameter while the others are kept constant. Furthermore, the curves depict the results of a second order polynomial fit in the software Origin (OriginLab, Northampton, MA, USA) to highlight the effects of changing the process parameters. The welding energy W_US_ in Figure 3a reveals the highest tensile shear forces at 2000 Ws with the best reproducibility, compared to the results at 1800 and 2200 Ws, respectively. A welding energy lower than 1800 Ws is not sufficient to achieve an adequate joint between AA5754 and AMZ4. In contrast, 2200 Ws leads to over-welding, in that connections are destroyed again by the sustained oscillations. A displacement amplitude u of 42.3 µm results in cracks in the aluminum sheet and consequently in a massive scatter of the tensile shear forces (Figure 3b). Insufficient values for the displacement amplitude as well as for the welding force F_US_ (Figure 3c) do not lead to adequate plastic deformations at the interface of the joining partners. In contrast, a welding force of 760 N causes sticking of the surfaces and restricts the relative motion, resulting in lower tensile forces than at the optimum welding force. With the general conditions regarding materials, geometries and the entire setup, only a very limited process window is available. The parameters are interdependent and in a suitable combination; only one specific value is found to be appropriate per parameter. Suitable process parameters for the AA5754/AMZ4 joints with the highest tensile shear forces of F_TS_ = 4509 ± 174 N are the welding energy W_US_ = 2000 Ws, the displacement amplitude u = 41 µm and the welding force F_US_ = 740 N. All further experiments use these parameters as the benchmark. Figure 4 shows images of successfully welded samples. The typical imprint of the sonotrode surface with the pyramidal profile marks the weld spot on the aluminum sheet (Figure 4a), while a slight discoloration is visible on the lower surface of the AMZ4 (Figure 4b), but no mechanical traces.

**Figure 5:**
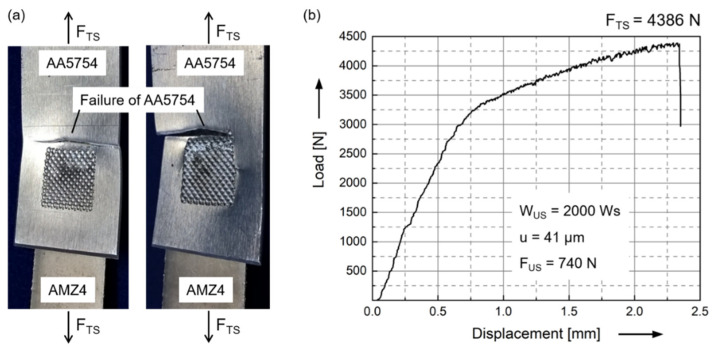
(**a**) Tensile shear tested AA5754/AMZ4 joints revealing the failure of the AA5754 sheet parallel to the edge of the weld spot. Process parameters: welding energy W_US_ = 2000 Ws, displacement amplitude u = 41 µm and welding force F_US_ = 740 N. (**b**) Characteristic load–displacement diagram of a tensile shear test of an AA5754/AMZ4 joint. Both elastic strain and plastic deformation occur, revealing the PLC effect that is typical for these aluminum alloys.

Tensile shear tests of AA5754/AMZ4 joints with suitable parameters result in a failure of the Al sheet (Figure 5a). Due to the overlapping joint, stress concentrations occur at the two edges of the weld spot, which are oriented perpendicular to the tensile direction. Since both the strength of AMZ4 and the joint strength are higher than the strength of AA5754, the highest stressed area is located in the aluminum sheet directly adjacent to the weld spot. In addition, the penetration of the sharp pyramids of the sonotrode into the Al sheet leads to a reduction of the cross-sectional area at the weld spot. The crack nucleates in the highest stressed area, directly at and parallel to the edge of the weld spot, and initiates the failure of the crystalline aluminum sheet. Thus, the structure exhibits plastic deformation behavior. In terms of an application, this would be advantageous because possible damage to the structure could be detected before failure. The load–displacement diagram of the tensile shear test reveals typical elements of the stress–strain diagram of AA5754 (Figure 5b). The yield strength of the Al sheet of 175 MPa times its cross-sectional area of 20 mm^2^ corresponds to the change in slope in the load–displacement diagram (approx. 3500 N) and the onset of increased plastic deformation. For a 5000 series aluminum alloy, the Portevin–Le Chatelier (PLC) effect is typical for deformations in the plastic regime so that the serrated curve can be seen in the load-displacement diagram [39].

**Figure 6:**
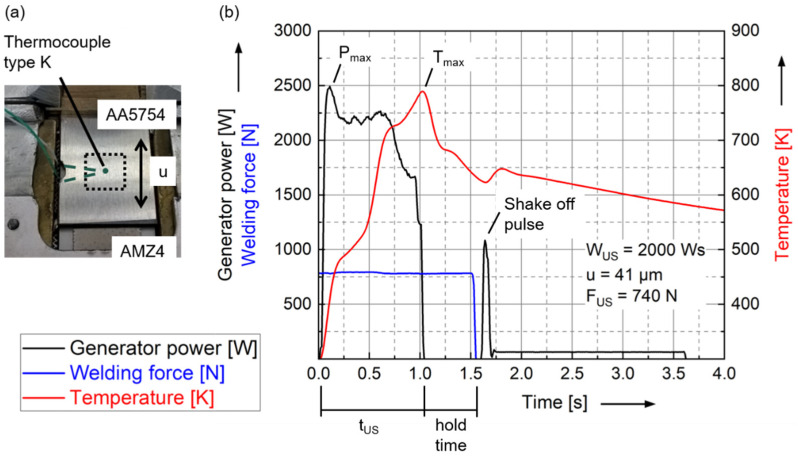
(**a**) Schematic illustration of the position of the thermocouple for temperature measurement at the interface in the center of the weld spot. (**b**) Characteristic curves of generator power, welding force and temperature measured with the thermocouple over time for AA5754/AMZ4 joints. The maximum temperature T_max_ at the weld spot of 807 ± 25 K is reached at the end of the welding time t_US_ of 0.96 ± 0.06 s.

The temperature characteristics of the welds are investigated using a NiCr-Ni thermocouple (type K) and an infrared camera in separate tests. The thermocouple is placed at the center of the weld spot at the interface of the two materials, as shown in Figure 6a. The thermocouple is introduced into the setup by removing a minimal amount of material from the aluminum sheet. Figure 6b shows typical curves for the generator power [W], the welding force [N] and the temperature measured with the thermocouple [K] over time. The welding time t_US_ observed is 0.96 ± 0.06 s, which is within a reasonable range for ultrasonic metal welding. The peak generator power P_max_ of 2639 ± 81 W is observed right at the beginning of the welding process to put the entire system, including the Al sheet, into the oscillating state and up to the nominal displacement amplitude. The peak temperature value T_max_ at the weld spot is 807 ± 25 K and is reached at the end of the weld. As long as energy is introduced in the form of oscillations, the temperature at the interface increases. The distribution of the measured values could be caused by the thermocouple in particular, as deviations could occur in shape and position. The welding time t_US_ is followed by the hold time, during which the sonotrode no longer oscillates but continues to press the joining partners together with the welding force F_US_. After the hold time, the machine lifts the sonotrode from the joining partners with a shake-off pulse. This pulse is necessary for the coupling surface to detach from the joining partner on the sonotrode side. When welding the soft aluminum alloy, the pyramids of the coupling surface penetrate into the aluminum sheet, and this form fit ensures the transmission of the oscillations. However, this also leads to adhesion of the aluminum to the sonotrode and, as a consequence, possibly to the destruction of the welded joint during lift-off. As long as the sonotrode is still in contact with the aluminum sheet, the renewed energy input in the form of vibrations causes the temperature to rise again.

**Figure 7:**
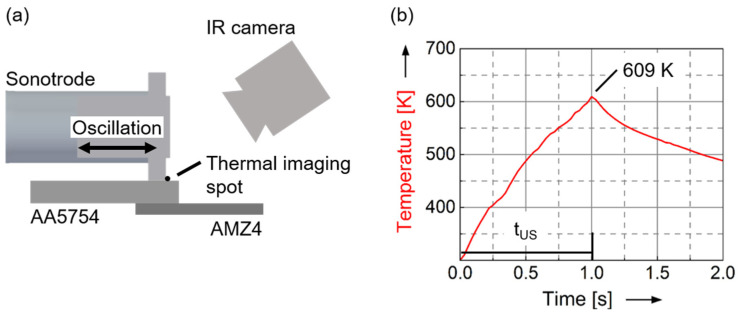
(**a**) Schematic illustration of the temperature measurement setup for infrared imaging. The thermal imaging spot is located at the center of the weld spot between the tip and the aluminum sheet. (**b**) Evaluation of the thermal imaging spot. The maximum temperature is measured 609 K after a processing time of 1 s.

The presence of a thermocouple at the interface can adversely affect the results, as it implies a change in the process conditions. It could have an influence on the temperature development during the welding process. In particular, verification of the temperature profile is essential, since values are reached for a short time, which could lead to crystallization of the AMZ4. Therefore, some experiments are performed with infrared imaging and normal conditions at the interface. Thermal data is collected in the direction of the oscillation, and the center spot is evaluated in the gap between the tool and the aluminum sheet, shown in Figure 7a. The results show a qualitatively comparable curve with a peak temperature of 609 K (Figure 7b). Due to the measurement at the sheet surface, the temperatures are approx. 200 K lower than at the interface.

**Figure 8:**
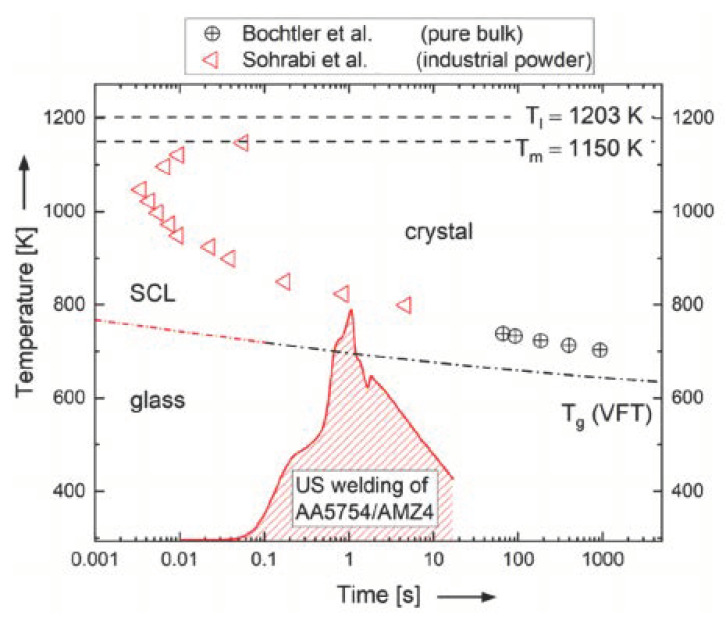
Isothermal time–temperature–transformation (TTT) diagram of AMZ4, based on [28,40,41,42]. The dashed lines correspond to the solidus temperature, T_m_, and the liquidus temperature, T_l_, which are both fixed temperatures upon heating (time-independent). The dashed–dotted line represents the time for the glass transition temperature T_g_, at a given temperature. It is approximately the structural relaxation time at a given temperature and therefore increases with decreasing temperature. Its temperature dependence can be well expressed with a Vogel–Fulcher–Tammann equation (VFT) [46]. The crystallization times at a given temperature are represented by red triangles (Sohrabi et al.) [40] and crossed circles (Bochtler et al.) [42]. The experimentally detected temperature profile of the ultrasonic (US) welding process is plotted as a red solid line and it does not reach the TTT for crystallization of AMZ4, rather it only reaches for a short time the supercooled liquid (SCL).

Figure 8 shows the isothermal time-temperature-transformation (TTT) diagram of AMZ4, which is experimentally determined in [28,40,41,42]. Here, the onset temperatures of crystallization T_x_ are plotted as a function of time (symbols) and follow the classical c-shape dictated by the nucleation theory [43]. The glass transition temperature T_g_ (dashed-dotted line) is rate-dependent, and for bulk metallic glasses is connected to vitrification kinetics [44,45] that follow a super-Arrhenius behavior well described with the Vogel–Fulcher–Tammann equation (VFT) [46]. The ultrasonic (US) welding process temperature profile that is measured in situ with a thermocouple (see Figure 6) is added into Figure 8 as a solid red line. Even if it is a simplification to use an isothermal TTT diagram to analyze a dynamic process, it appears that thermal load during ultrasonic welding is high enough for AMZ4 to undergo glass transition and to reach the supercooled liquid region (SCL), which allows the AMZ4 to flow and enable the joining with the Al-alloy. On the other hand, it is low and short enough to avoid devitrification via crystallization of AMZ4. In fact, the temperature profile detected in the joined region during ultrasonic welding does not reach the onset of crystallization, represented by the opened red triangles [40].

**Figure 9:**
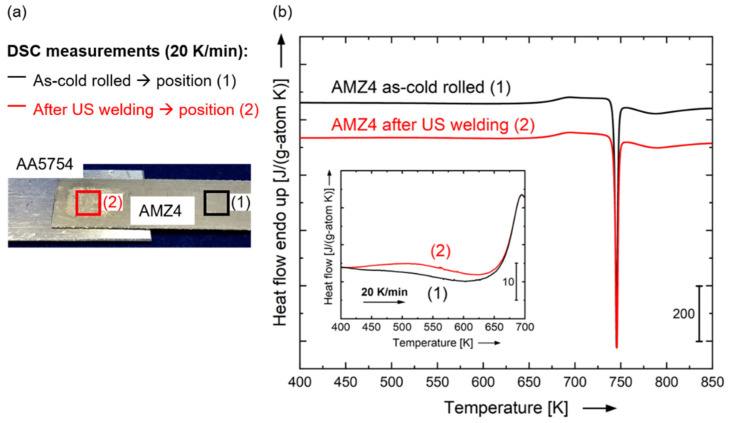
Differential scanning calorimetry study of AMZ4 before and after ultrasonic welding. (**a**) Photograph of the welded sample showing the positions from which the samples for the calorimetry are cut, where (1) indicates AMZ4 material far away from the joint, which represents AMZ4 in the as-rolled state, and (2) the AMZ4 part subjected to ultrasonic (US) welding. (**b**) Subtracted heat flow scans for the as-rolled AMZ4 (1) and AMZ4 after US welding (2). ΔH_x_ is the heat of crystallization associated with the area of the first exothermic peak, which is obtained by integrating the peak. The inset shows the magnified heat flow signals in the low-temperature regime just before the sharp increase at the glass transition. The welded sample shows a smaller (less negative) heat release due to relaxation than the as-rolled sample, because it already encountered some degree of relaxation during the welding process.

Figure 9 reports the differential scanning calorimetry (DSC) subtracted heat flow scans performed with AMZ4 material that is taken from the joint region (AMZ4 in as-welded conditions (2)) and far away from the joint (AMZ4 still in as-cold rolled conditions (1)). The two DSC signals are very similar with identical glass transition (step in heat flow with onset at T_g,onset_ = 658 K) with a main first sharp crystallization peak at 745 K and a second smeared out peak at higher temperature. The heats of crystallization associated with the first crystallization event are very similar to each other within the experimental error (typically in the order of ±0.3 to 0.4 kJ/g-atom). The fact that the crystallization enthalpy ΔH_x_ after welding measured by DSC (2.77 kJ/g-atom) is comparable to the as-rolled condition (2.61 kJ/g-atom) confirms what Figure 8 has anticipated, i.e., that the ultrasonic welding does not induce any crystallization of AMZ4.

These DSC up-scans are plotted at higher magnification in the inset, rendering a better view of the low-temperature signals up to the end of the glass transition step. In both DSC scans, an exothermic broad signal starts at low temperature. This event is visible only at high magnification in the inset plot. This is an exothermic event below T_g_ that corresponds to structural relaxation phenomena (physical aging), giving information about the thermal history of the specimen [47,48]. Cold rolling of the sample results in a continuous increase in the average shear band density and induces compressive residual stresses, and consequently, the measured exothermic heat released preceding the glass transition is increased [49]. Subsequently, the welding process causes a reduction of the relaxation enthalpy (area underneath the relaxation signal), resulting from the introduced heat into the sample, allowing partial structural relaxation to occur during welding. This is in good agreement with the measured processing temperature in the heat affected zone.

**Figure 10:**
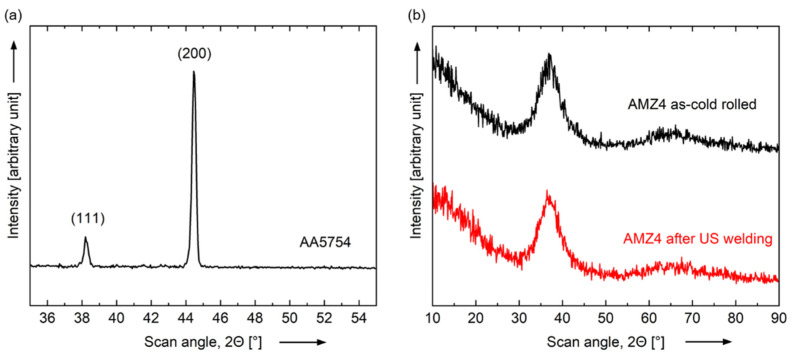
XRD patterns (CuKα radiation) of (**a**) AA5754 and (**b**) AMZ4 in as-cold rolled condition and AMZ4 after ultrasonic (US) welding with the welding energy W_US_ = 2000 Ws, the displacement amplitude u = 41 µm and the welding force F_US_ = 740 N. The 2Θ-scan of AMZ4 after US welding reveals the condition at the fractured surface of the weld spot after removing the aluminum sheet. The patterns of AMZ4 in as-cold rolled condition and AMZ4 after US welding are qualitatively the same and show no signs of crystallization of the metallic glass due to the welding process.

Further investigations on the influence of the welding process on the amorphous state of AMZ4 are carried out by X-ray diffraction analysis (XRD). Figure 10b shows the comparison of two surface scans of AMZ4 in as-cold rolled condition and AMZ4 after ultrasonic (US) welding. Both curves contain broad disperse peaks in contrast to the distinct sharp peaks of crystalline AA5754 in Figure 10a, which correspond to the (111) and (200) planes. All measurements are based on the same settings. The scan of AMZ4 after US welding is performed on the fractured surface of the weld spot after removing the aluminum sheet. Since this is the interfacial area between the amorphous and crystalline metals, the highest process temperatures are expected there. Both scans of AMZ4 in as-cold rolled condition and AMZ4 after (US) welding show a broad diffraction peak in the range of 25–40°, which is typical for an amorphous structure [16] and has been demonstrated in previous studies of AMZ4 [28]. Since both curves are qualitatively the same and no sharp diffraction peaks appear, the measurements indicate that AMZ4 is still in the amorphous state after welding [2,13].

**Figure 11:**
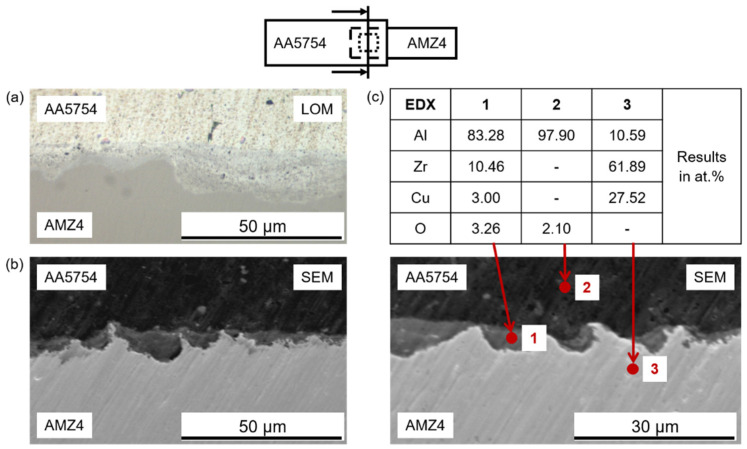
Cross-section morphologies of AA5754/AMZ4 interfaces at the weld spot and at the condition of the welding energy W_US_ = 2000 Ws, the displacement amplitude u = 41 µm and the welding force F_US_ = 740 N: (**a**) light optical micrograph (LOM), (**b**) scanning electron micrograph (SEM) and (**c**) SEM/EDX analysis accompanied by atomic percentage of the points 1, 2 and 3.

Further investigations include analyses by light optical microscopy (LOM) and scanning electron microscopy (SEM). Figure 11a shows an optical micrograph of a cross-section at the interface. No straight interface was visible; instead, there seemed to be a mechanical interlocking of both materials. The soft aluminum appeared to be pressed into the surface structure of the harder AMZ4, creating large surface area of contact. The SEM images confirms this observation (Figure 11b). The cross-sections are additionally characterized by EDX spot measurements. The results are displayed in atomic percentage in the table of Figure 11c. The elemental distributions show an apparent discontinuous “intermediate layer” between the Al alloy and the metallic glass. The EDX analysis (point 1) suggests that it is Al-rich and contains some Zr and Cu. However, the regions are too small to exclude that some of the AMZ4 was inside the excited area contributing to the signal, thus producing an apparent Zr and Cu content that is not existing. It is, in fact, unlikely that within the short processing time of a few seconds considerable mixing between the alloys takes place. It is therefore also possible that the contrast in the micrographs results from the fact that this material is heavily deformed aluminum, which is locally pressed into the metallic glass. The EDX analyses of the two alloys (points 2 and 3) fit well with nominal concentrations in the alloys.

The investigated process of ultrasonic metal welding of metallic glasses and crystalline alloys may be of particular interest for high-quality special tools. BMGs are predestined for functional surfaces that must be hard and wear-resistant. Due to the challenges of the manufacturing process, it makes sense to produce only the functional surface out of BMG. Residual components can be made from crystalline materials, which are easier to machine and less expensive. Welding creates a reliable and durable connection between components without the need for additional components or complex geometries. Medical tools could be a suitable field of application, and the service life of such tools could be extended by a very robust functional surface.

## 4. Conclusions

Ultrasonic metal welding enables the successful joining of crystalline AA5754 and amorphous AMZ4. The key factor is the short process in the solid state (AA5754) and supercooled liquid state (AMZ4) of both alloys. The following conclusions can be drawn:

- Optimization of the process parameters with the welding energy W_US_ = 2000 Ws, the displacement amplitude u = 41 µm and the welding force F_US_ = 740 N, results in Al/BMG joints that exhibit tensile shear forces of F_TS_ = 4509 ± 174 N, equal to the Al base metal. The joint itself is not the weakest link, as plastic deformation (PLC-effect) and failure occurs in the crystalline Al sheet.
- Considering the TTT diagram of the AMZ4, the thermal load during ultrasonic welding enables AMZ4 to undergo glass transition and to reach the supercooled liquid region (SCL), resulting in the joining of the two alloys without any crystallization in the BMG occurring during the welding process.
- Differential scanning calorimetry (DSC) reveals that the enthalpy of crystallization after welding is comparable to the as-rolled condition. This is due to the low and short thermal loading of the joining process, which prevents the crystallization of AMZ4. In addition, the released enthalpy of relaxation of the weld spot in the DSC is smaller than in the as-cold rolled condition, because the temperature rise above the glass transition and the subsequent quench of the weld spot leads to a more relaxed state as the as-rolled BMG sheet. Morphology stability and microstructural investigations by XRD, LOM and SEM/EDX reveal no evidence of crystallization of AMZ4 in the bulk material.
- Future investigations could achieve further improvement in joint performance through detailed parameter studies and through improved mechanical properties of the crystalline joining partners (e.g., AA7075, Ti64). The limits of ultrasonic metal welding for this combination of materials could be determined if the strength of the crystalline joining partner is large enough so that the joint fails at the weld interface during shear loading.

## Figures and Tables

**Table 1:** The results of monotonic tensile tests of AA5754-H22 according to the DIN EN ISO 6892-1 process A: yield strength (σ_y_), ultimate tensile strength (UTS), Young’s modulus (E) and ultimate elongation at fracture point (A). In addition, the results of the compression and 3-point bending test of AMZ4: yield strength (σ_yield_), maximum strength (σ_max_) and compression or bending modulus (E) for both cases.

**Specimen**	**Tensile Properties**					
	**σ_y_** **[GPa]**	**UTS** **[GPa]**	**E** **[GPa]**	**A** **[%]**		
AA5754-H22	0.178	0.250	70	13.5		
	**Compression Properties**			**Bending Properties**		
	**σ_yield_^compr.^** **[GPa]**	**σ_max_^compr.^** **[GPa]**	**E^compr.^** **[GPa]**	**σ_yield_^bending^** **[GPa]**	**σ_max_^bending^** **[GPa]**	**E^bending^** **[GPa]**
AMZ4	1.6	1.7	90	2.1	2.7	78

## Data Availability

The data presented in this study are available in the article.
